# P08 Overview of general antimicrobial prescribing guidance across NHS trusts in the United Kingdom: analysis of the Induction^™^ MicroGuide platform with a focus on hospital-acquired pneumonia (HAP) disease definitions

**DOI:** 10.1093/jacamr/dlad066.012

**Published:** 2023-06-14

**Authors:** Tom Ashfield, Stephen Hughes, Ioannis Baltas, James Amos, Mineli Cooray, Luke S P Moore

**Affiliations:** Pfizer Ltd, UK; Chelsea & Westminster NHS Foundation Trust, UK; Imperial College Healthcare NHS Trust, UK; Pfizer Ltd, UK; Pfizer Ltd, UK; Chelsea & Westminster NHS Foundation Trust, UK; Imperial College Healthcare NHS Trust, UK; Imperial College London, London, UK

## Abstract

**Background:**

Effective antimicrobial prescribing and stewardship for acute severe infections require prescribers to use empirical guidance for treatment decisions implemented prior to diagnostic confirmation. Many United Kingdom (UK) National Health Service (NHS) trusts provide such guidance via the Induction^™^ MicroGuide platform. We explored the availability of general antimicrobial stewardship (AMS) principles and more specifically, hospital-acquired pneumonia (HAP) guidelines within this platform.

**Methods:**

Acute NHS trusts in the UK (nine commissioning regions) with general antimicrobial use guidelines uploaded on MicroGuide were analysed for the inclusion of key sections (sepsis management, AMS, IVOS [IV to oral switch], antifungal guidance, OPAT [outpatient parenteral antimicrobial therapy] and critical care) and time since last update. HAP guidelines were assessed across a number of sections including disease classification and severity definitions. Guidelines were accessed over 12 days (21 October–2 November 2022). Data were not missing for any trust.

**Results:**

Overall, 115 UK trusts used the Induction^™^ MicroGuide platform to host their antimicrobial guidelines. Four trusts had separate guidelines for different hospitals within that trust (two each); the final number of guidelines analysed was 119. Eleven hospitals (9.2%) included all key general guideline sections; most guidelines included sepsis management (*n* = 117/119; 98.3%) and AMS (*n* = 112/119; 94.1%) sections. IVOS (*n* = 99/119; 83.2%) and antifungal guidance (*n* = 83/119; 69.7%) sections were also common. OPAT guidance was less common (*n* = 56/119; 47.1%) and critical care sections were mostly absent (*n* = 27/119; 22.7%). All hospitals included ≥1 HAP guideline section. HAP disease classification included time to onset after admission (*n* = 12/119; 10%), severity (*n* = 69/119; 58%), a combination of both (*n* = 30/119; 25%) or no classification (*n* = 8/119; 7%). HAP severity definitions varied significantly across hospitals, with some including terms like complex/critical/life threatening, and others including resistance, advanced age, severity scores and the presence/absence of sepsis or requirement for intubation or mechanical ventilation. Ventilator-associated pneumonia (VAP) guidance was available for 37 (31%) of hospitals; 82 hospitals did not have separate sections for VAP guidance (69%). About half the number of hospitals (*n* = 62/119; 52%) updated their guidelines ≤2 months prior to analysis; 97% updated them within ≤1 year prior to analysis.

**Conclusions:**

General guideline sections identified as key to AMS and patient safety are readily available in most NHS Trusts using MicroGuide. The majority of NHS Trusts have guidelines available for HAP; however, disease and severity definitions vary considerably. Achieving consistency in disease definitions and thresholds for treatment across trusts is likely to be beneficial for AMS efforts.

